# Public Policy Measures to Increase Anti-SARS-CoV-2 Vaccination Rate in Russia

**DOI:** 10.3390/ijerph19063387

**Published:** 2022-03-13

**Authors:** Dmitry V. Boguslavsky, Natalia P. Sharova, Konstantin S. Sharov

**Affiliations:** Koltzov Institute of Developmental Biology of Russian Academy of Sciences, 26 Vavilov Street, 119334 Moscow, Russia; boguslavsky@rambler.ru (D.V.B.); const.sharov@mail.ru (K.S.S.)

**Keywords:** vaccination rate, conspiracy beliefs, QR codes, vaccination certification, COVID-19 pandemic, feedback of population

## Abstract

The total vaccination rate remains relatively low in Russia as of March 2022 (around 55%, with around 20% in some regions). In the paper, we study the reasons for it. We communicate the results of our survey aimed at detecting reasons for the relatively low anti-SARS-CoV-2 vaccination rate in Russia (47.1% as of mid-January 2022) and suggest potential measures to increase the level of confidence in the Russian vaccination campaign. A total of 14,310 users exhibited interest to participate in the research (16.84% of the total number of invitations sent in the Russian social network VKontakte). After the sample set repair, only 5822 (40.68% of those who agreed to participate) responses were suitable for the research, and they composed the final set. The age range of the respondents was 16–51 years old (y.o.) with a mean of 29.1 ± 10.6 y.o. The proportion of the female gender in responses was 44.23%. A total of 2454 persons (42.15%) expressed their hesitant, cautious, or negative attitude towards vaccine uptake. Of the 2454 persons with cautious attitude towards vaccination, only 928 (37.82%) were concerned about the quality of the Russian vaccines. A total of 1323 individuals (53.91%) supported one or more conspiracy beliefs. A total of 5064 (86.98% of the whole set) showed cautious or negative attitude towards the planned introduction of a nationwide system of vaccination certification/verification based on QR codes. The main social factors that hinder the Russian vaccination campaign are: vexation over the lack of desire of officials to receive feedback from the general population regarding vaccination, wide support for conspiracy beliefs, and controversy over the QR code-based digital system. To elevate the vaccination rate in Russia, the following steps may be taken: social encouragement of those who support vaccination, increase in transparency of the vaccination campaign, acceptance of both digital and paper vaccination certificates, increase in participation of society in vaccination-related discussions, public disclosure of vaccine composition, and avoidance of excessive digitalization of data in the vaccination campaign.

## 1. Introduction

### 1.1. Current Level of Anti-SARS-CoV-2 Vaccination in Russia

Despite recent successes in the anti-SARS-CoV-2 vaccine development and the fact that the vaccination in Russia has already started in late 2020, the total Russian vaccination rate remains comparatively low. Currently (as of 4 March 2022, the date of revising the paper after the first round of reviewing), the total number of cases since the beginning of the pandemic is 16,685,850; the change in the number of new COVID-19 cases in Russia, 89,174; the change in the number of new hospitalized people, 9849; and the change in the number of new convalescent people, 160,507 [1]. The mortality rate is 1.17% [1]. The main Russian vaccines used are (1) Gam-COVID-Vac (Sputnik V), (2) EpivacCorona, and (3) CoviVac [1]. The proportion of those vaccinated with Gam-COVID-Vac among all vaccinated persons in Russia may be estimated as 85–90% [2].

Data on the current vaccination rate in Russia obtained in different sources are somewhat contradictory. The official online resource devoted to the struggle with COVID-19 “Stopcoronavirus” [1] provides a figure of 52.5% of the total Russian population as of 15 January 2022. The most authoritative independent statistical agency Gogov reports 47.1% as of 15 January 2022 [2].

There are substantial discrepancies in the vaccination rate across different regions of Russia. According to the vice prime minister’s report, four Russian regions may vaunt the level over 80% [3]. According to data taken from independent sources [2,4], while St. Petersburg and most of large cities and densely populated regions reached figures of 50–70% as of January 2022, the rate in little-inhabited regions is often as low as 15–30% (e.g., North Caucasian republics and some other southern Russian provinces) [2,4]. The vaccination rate in Moscow was below 50% as of 15 January 2022 [2].

### 1.2. Prevalent Reasons for Low Anti-SARS-COV-2 Vaccination across the World

There are several most common factors that hinder anti-SARS-CoV-2 vaccination in different countries. Among them, the researchers notice contradictory statements of federal and local authorities about vaccination campaign planning, vaccine prioritization/allocation, and time boundaries [5,6]; lack of the authorities’ desire to study the feedback of the general population [7,8]; mistrust of official information about the vaccines available [9]; and antivaccination (“anti-vaxxer”) social movements [10] in high-income countries. In low- to middle-income countries, they note vaccine shortages [11,12], perpetual distrust of almost any governmental initiatives [13], low level of healthcare literacy [14], lack of social/economic stimuli to be vaccinated [15], inequalities in access to vaccines [16,17], geographical remoteness of large parts of a population from vaccination sites [18], and poor informational/media coverage of vaccination campaigns [19]. Besides, the enormous surge of COVID-19-related conspiracy ideas hampers vaccination in both types of countries [20].

There is no unity in opinions on the low vaccination rate in Russia yet. In May 2021, Alexander Gorelov, deputy director of the Central Institute of Epidemiology of Rospotrebnadzor, one of the top officials engaged in the Russian anti-COVID-19 campaign, blamed the Russian antivaccination movement [21], while in October 2021, Gleb Kuznetsov, head of the Expert Council for the Institute of Social Investigations, cast doubts that supporters of the Russian antivaccination movement play any considerable role in the low vaccination rate in Russia [22]. In its article, the Associated Press agency related the Russian low vaccination level to distrust of authorities and lack of confidence in official data on vaccination and vaccines [23]. Different conspiracist ideas might also be the factors of impediment to the vaccination progression in Russia, as some reporters suggested in spring 2021 [24,25,26].

### 1.3. COVID-19-Related Conspiracy Theories as Obstacles for Vaccination

In many countries, low anti-SARS-CoV-2 vaccination levels are currently associated with social resistance partly based on beliefs in hidden social and political forces that allegedly pursue their own mainly pernicious goals through the ostensible struggle with the COVID-19 pandemic [27,28,29]. COVID-19-related conspiracy theories are known to have made serious obstacles in various countries with quite different cultural heritages, religions, ethnic compositions, economic developments, and levels of income: in Canada [30], Croatia [31], Germany [32,33], Ghana [34], India [35], Jordan [36], Kuwait [37], Pakistan [38], People’s Republic of China [39], Poland [40], Saudi Arabia [41], Turkey [42], Ukraine [43], the United Kingdom [44,45], and the United States [46,47].

Kostas Gemenis emphasized that pandemic-related conspiracy beliefs, irrespective of the country of origin, are alike and form stable and coherent narratives [48]. Daniel von Wachter performed perhaps one of the most profound social analyses of COVID-19-related conspiracy theories and found that there should be a distinction between “ungrounded” conspiracy beliefs (e.g., SARS-CoV-2 is a special biological weapon) and “rational” conspiracy beliefs (i.e., the system of ideas and corresponding heath behavioral responses of a population) by means of which people try to find logic in administrative steps taken by authorities in the COVID-19 situation and explain them somehow [49]. Many researchers stress that COVID-19-related conspiracy beliefs are spread like an infodemic because of social network communication [50,51,52,53,54,55,56,57,58,59]. A considerable part of conspiracist ideas in different countries is devoted to COVID-19 vaccination and prevention measures associated with using QR (quick response) codes [60,61,62]. There are no sociological studies of this problem in Russian society yet.

There is no agreement of scientists yet on the role of trust in conspiracies in fettering the Russian anti-SARS-CoV-2 vaccination campaign. As we shall show, in Russia the QR code controversy both plays an important role in supporting antivaccination conspiracist sentiments in the population and presents a technical obstacle to implementing vaccination certification and control.

### 1.4. Specific Aim

In the paper, we present and analyze the results of our survey among Russian social network users who agreed to participate in this vaccination-related research to find a rationale for skepticism about vaccine uptake in Russia. We discuss the reasons for the social opposition to anti-SARS-CoV-2 vaccination and suggest several potential measures to increase the level of confidence in the Russian vaccination campaign and the federal and regional authorities and, therefore, to elevate the level of the collective anti-SARS-CoV-2 immunization in Russia effectually.

## 2. Methods

### 2.1. Description of the Survey

Initially, an invitation was sent to 85,000 social network account holders (social network VKontakte (“In Contact”)) who demonstrated their interest in discussing the anti-SARS-CoV-2 vaccination campaign in Russia during the year 2021 (based on the posts placed). A total of 85,000 participants were chosen based on the number of Russian regions (85) (1000 invitations per region), and the invitation submission was done in VKontakte automatically through the proprietary software. Of those who were sent the invitation, 14,310 users exhibited interest to participate in the research (16.84% of the total number of invitations). They responded to the questionnaire (Supplementary Material) using VKontakte online capabilities and a proprietary Silverlight applet.

After the sample set repair, only 5822 (40.68% of those who agreed to participate) responses were suitable for further research, and they composed the final set.

Suitability was defined as follows. A suitable response is a completed questionnaire without spoilt, incredible, or strange fields (Online Supplementary Material). If there was no clear response in the questionnaire to all the questions, we tried to obtain the necessary information from the social network profile in question by the content analysis of the posts placed in 2021. If we failed here, such a questionnaire was deemed unsuitable for further study.

We eliminated spoilt and strange responses and responses with low credibility in algorithmic (stage 1) and manual (stage 2) ways.

1. (Algorithmic) First, a program screened through the database and carried out the simplest linguistic analysis. It removed all responses in which at least one field was blank or at least one field did not contain at least one intelligible word in Russian.

2. (Algorithmic) Second, a program screened through the database and eliminated all responses with (1) at least one field of field nos. 4, 6, and 9 different from {YES, NO, DIFFICULT TO SAY}; (2) at least one field of field nos. 2, 5, and 8 different from {YES, NO}; (3) field no. 9 different from {VOLUNTARY, COMPULSORY, DIFFICULT TO SAY}.

3. (Manual) Finally, manual content analysis of field nos. 1, 3, and 7 was performed. If a questionnaire contained any of these fields that were unintelligible, strange (irrelevant), or empty, this questionnaire was place “on hold.” If manually screening the account in question could obtain an answer, the questionnaire was completed by the authors on the basis of the information contained in the social network profile. If we could not obtain an answer, we discarded the questionnaire.

The complete description of the set repairing methodology used may be found in our recent work where a similar surveying technique was applied for another research purpose [63].

### 2.2. Time of Study

The main results were obtained in the interval 1 September 2021 to 25 January 2022.

### 2.3. Software

Origin 8.1 (OriginLab, Northampton, MA, USA) was used for modelling, statistical calculations, and visualization. Microsoft Visual Studio (Microsoft Corp., Redmond, WA, USA) was used for computing proprietary screening applications.

### 2.4. Geographical Diversity

Responses of people from all 85 Russian regions were included in the research. However, the representativeness of different regions was different. That depended on the social network activity and desire to participate in the research.

## 3. Results and Discussion

### 3.1. Different Factors of Low Vaccination Rate in Russia

Table 1 presents the main results of the survey and some demographics of the respondents.

Of the 5822 people surveyed, 2454 (42.15%) expressed their hesitant, cautious, or negative attitude towards vaccine uptake (Table 1). Of the 2454 persons with cautious attitude towards vaccination, only 928 (37.82%) were concerned about the quality of the Russian vaccines, while the rest were occupied by the social implications of vaccination (“a new order of things”), be they connected to considerations of economy, social welfare, education, conspiracy theories, or other factors. We must recognize that the main reasons for the low vaccination rate in Russia are social, not healthcare related.

Among the social factors of low vaccination rate, we found the most common ones: concerns about digital innovations in the education, social welfare, and healthcare sectors caused by the pandemic; vexation over the lack of administrative desire to receive feedback from the population; controversy over the forthcoming federal QR code-based system of vaccination verification; and support for conspiracy beliefs (Table 1). Doubts about the safety of Russian vaccines, concerns about equality in vaccine allocation, preference for foreign vaccine over the Russian ones, and confirmed antivaccination attitudes (irrespective of vaccine or infectious disease) play a much smaller role in overall vaccination hesitancy in Russia.

### 3.2. Role of COVID-19-Related Conspiracy Theories in Vaccine Uptake Hesitancy in Russia

Of the 2454 persons who declined to get vaccinated, demonstrated caution towards vaccination, or wavered, 1323 individuals (53.91%) responded that they did support one or more beliefs that are usually categorized as conspiracy theories. This group of population composes 22.72% of the total sample set. It is a high proportion. It is interesting that if a person believes in COVID-19-related conspiracies, he or she does not do it selectively; that is, he or she usually believes in many conspiracies all at once (Table 1).

### 3.3. QR Code Controversy

Whereas many conspiracist beliefs are associated with QR codes, the QR code controversy in Russian society goes much beyond the area of trust in conspiracies.

At the beginning of September 2021, government media announced that the Russian government was elaborating a bill about QR codes as a surveillance, controlling, and informational technique for combating the COVID-19 pandemic. It was said that this system would contain digital vaccination certificates and might facilitate the Russian vaccination campaign. In autumn 2021, the beginning of the parliamentary discussion of this bill was scheduled in February 2022. There were references to positive experiences of a number of countries that applied QR codes or similar technological tools for effectual contact tracing, disease prevention, and vaccination facilitation.

The QR code controversy represents a separate factor that may cause serious—perhaps most serious—problems in the Russian vaccination campaign. Therefore, a brief survey on applying QR codes or similar technological contrivances in the struggle with COVID-19 in several countries may be useful here. It demonstrates different outcomes in different societies.

A number of authors emphasize effective capabilities of a contact tracing/social monitoring/vaccination certification system based on QR codes: rapidity [64], convenience [64], epidemiological safety [64,65], effectiveness of ensuring social distance compliance [65], suitability for telemedicine services [66], and safety of remote delivery of a patient’s information [66].

The first country to introduce a nationwide system involving COVID-19-related QR codes was China. In summer 2020, this system was tested in Fujian province [67], while in 2021, it was employed on the national scale [68,69]. For the Chinese realities, a QR code system included three colors of QR codes (red: active spreader; yellow: suspicious or convalescent person; green: not infected). It demonstrated good results in effective tracing contacts, organizing urban and periurban infrastructure, ensuring quarantine measures, and finally facilitating anti-SARS-CoV-2 vaccination as an indirect consequence. Some other Far Eastern and Southeast Asian countries (e.g., Republic of Korea [70], Taiwan [71], and Singapore [72]) also showed positive results in applying QR codes for different COVID-19-related epidemiological measures, including issuing and controlling/verifying digital vaccination certificates. Singapore solved the problem of uneven access of population to smartphones radically. Singaporean authorities applied a nationwide system of digital Bluetooth-enabled wearables TraceTogether [72]. It contains QR codes and other technologies that help to locate the position of every person to within 0.5 meter, monitor his/her health status, report to the health authorities in 24/7 mode, ensure timely vaccination, and disseminate new information about governmental measures against the pandemic.

However, there is also a different view on the abilities and efficacy of QR code-based systems. Liu and Komissarov hypothesize that the success of the QR code-based system in China is explained by the strong adherence of Chinese society to the Confucian ideals of “common deal” [73]. These authors doubt that this system could reach the same success in the Western world (e.g., in the USA) [73]. Horgan et al. also points out the low applicability of Far Eastern practices of QR code application in curtailing the pandemic in more “democratic” societies (e.g., United Kingdom) [74].

Min-Allah et al. demonstrate that in real life, a QR code-based or similar digital system may give poor results even in its core functionality (i.e., contact tracing) due to technological errors, problems with Internet connection, low digital literacy of the population in many low- to middle-income countries, and low level of smartphone use [75]. Oleg Donskikh argues that applying QR codes is dubious from a moral viewpoint, as it may lead to strengthening self-isolation, new stratification of society, and decreasing the quality of education and healthcare due to mass switch to distant online practices [76]. Several researchers stress that using QR codes for curtailing the pandemic must be highly selective and not universal. Thong et al. [77] and Cook et al. [78] suppose that a QR code-based system may be used for telemedicine services only. Khan et al. [79] argues that it may be functional only for needs of distant work to ensure uninterrupted processes. Wolfgang Sassin [33,80] and Oleg Donskikh [76] admit the effectiveness of a QR code-based system for the control of transboundary relocation and its awkwardness in use within a country’s borders. These authors agree that in many areas (e.g., contact tracing, relocation control, face control, and vaccination status control) a QR code-based system may cause problems.

As our research shows, a substantial part of the Russian population considers the planned introduction of a QR code-based system in Russia negatively: 94.03% of the group of antivaccination activists and 86.98% of the total sample set of 5822 persons (Table 1). One can see that many Russian people who oppose the introduction of QR codes on the national scale do not support any conspiracy beliefs, nor exhibit confirmed antivaccination sentiments.

Most importantly—and unexpectedly—the relentless governmental attitude towards introducing a national system of vaccination verification based on QR codes in September 2021– January 2022 may have antagonized many potential vaccination supporters, against vaccine uptake. Around one quarter of the surveyed persons recognized that the introduction of a national QR code-based system may avert them from potential vaccination (Table 1).

**Figure 1:**
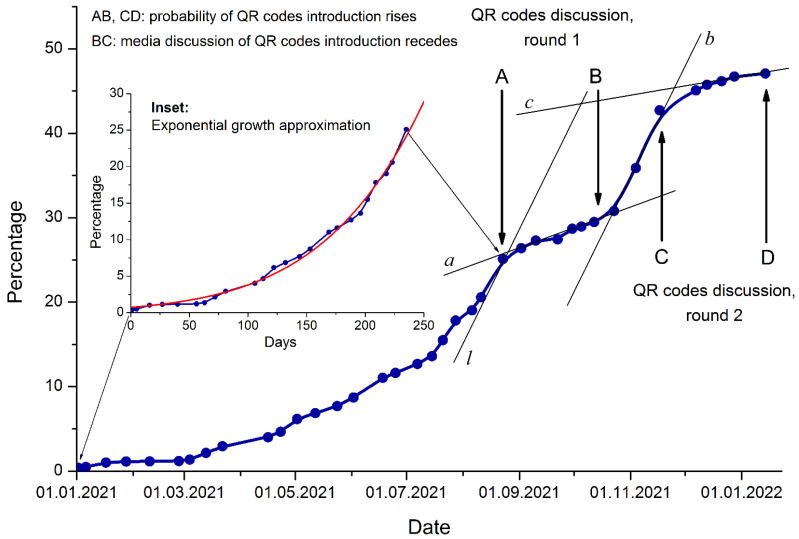
Anti-SARS-CoV-2 vaccination progression in Russia. Main figure: Percentage of the vaccinated Russian population (vaccination rate) since the beginning of 2021 thus far. The data are taken from the Gogov statistical agency [2]. Only fully vaccinated persons (at least two doses) are considered. Inset: Exponential growth approximation of the first segment OA (1 January 2021–23 August 2021) (red curve), with *x*-axis graduated in days since 1 January 2021. Two rounds of governmental discussion of the QR code program are shown as segments AB and CD. See the details and statistical treatment of the approximations in the text.

Figure 1 shows the vaccination progression in Russia depending on time.

There are four clearly distinguishable segments of the line: OA (the initial stage up to the end of August 2021), AB (the first slowdown: 23 August–20 October 2021), BC (a new acceleration: 20 October–25 November 2021), and CD (the second slowdown of vaccination rate: 25 November–15 January 2022). The initial stage OA shows exponential growth of the vaccination rate:

$$V=e^{at^{2}+bt+c},$$

where *V* is vaccination rate, percent; *t* is time, days since 1 January 2021; and coefficients are the following: *a* = –1.175∙10^–5^ ± 0.621∙10^–5^, *b* = 0.0177 ± 0.0022, and *c* = –0.31 ± 0.19. The quality of approximation is as follows: reduced *χ*^2^ = 0.3445, residual sum of squares *RS_sq_* = 7.2347, and adjusted coefficient of determination *R_adj_*^2^ = 0.9936 (a good approximation).

However, since September 2021 (segment AD) the vaccination rate experienced two periods of substantial slowdowns (segments AB and CD), and it was far from exponential growth. During periods AB and CD, the Russian media were spreading torrents of information on the forthcoming introduction of the QR code-based system with relentless attitude of the Russian government that was seemingly not ready to listen to the criticism at that time. We performed day-to-day content analysis of news releases in major governmental media during this period. We surveyed TV channels: 1TV, Russia 1, Russia 24, TVC, and NTV. We took into account the quantity of QR code-related news (*q*) and time duration (*t*). Then we analyzed the variable *T* = *q* · *t* for each day in this period for the five TV channels concerned. The higher was T, the greater was media pressure. During the period BC, there were almost three times fewer media messages about the QR code system on the main TV channels. During this time, the government collected data about the experience of several Russian regions that decided to introduce the QR code system in test mode ahead of the federal law. For instance, the first region of Russia to introduce a local QR code-based system of vaccination verification was Tatarstan. The measure was launched on 22 November 2021, and epidemiologically risky mass gatherings, street protests, and even social unrest immediately ensued.

The Russian federal government and local authorities faced strong opposition from the general population, a number of prominent public figures, several research institutions, nongovernmental organizations (NGOs), and not-for-profit organizations (NPOs) regarding introducing the QR code-based system (segment BC). Nonetheless, the federal government decided to stick to the QR code project on the federal level in mid-November 2021. This information was announced in the media, and the vaccination rate dropped again (segment CD). On 14 January 2022, at the time when this article was being written, there was a news release according to which the bill about launching the QR code-based vaccination certification/verification system on the federal scale would be finally withdrawn from the Russian Parliament (Duma) and returned to the government for improvement due to a multitude of critical remarks and responses. In February 2022, there would be no parliamentary voting.

We see that the social mood related to the feasible introduction of the QR code-based system of vaccination certification/verification influenced the vaccination rate in Russia as a negative feedback: the greater was the governmental pressure, the lower was the rate of vaccination. To assess the degree of dampening the readiness to vaccine uptake, we may measure the angles that lines *l, a, b,* and *c* form with the positive direction of the x-axis. The tangent line l to the exponential curve at point A is nearly parallel to line b (the second acceleration of the vaccination rate). Then the ratio of tangents
tan λ : tan α : tan γ = tan (*l* to *x*) : tan (*a* to *x*) : tan (*c* to *x*) = tan β : tan α : tan γ = tan (*b* to *x*) : tan (*a* to *x*) : tan (*c* to *x*) = tan 63° : tan 18° : tan 10° = 1.9626 : 0.3249 : 0.1763 = 11.13 : 1.84 : 1
reflects the ratio of the speeds of vaccination at segments OA (in point A) (as well as BC), AB, and CD. We see that QR code-related administrative uncertainty lowered the pace of vaccination 6 times in autumn 2021 and almost 12 times in winter 2021–2022.

**Figure 2:**
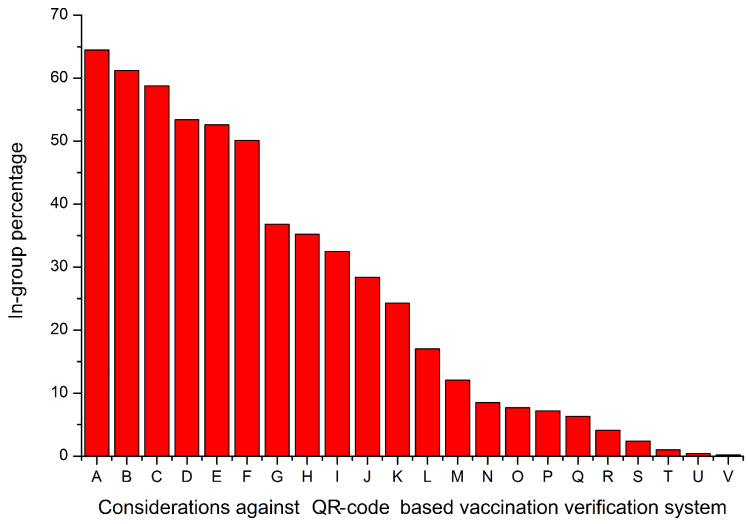
The frequency of occurrence of the main objections to introducing the nationwide system of vaccination certification/verification based on QR codes found in our survey. In-group percentage shows parts of a group of 5064 persons cautious about the planned QR code-based system. The legend is provided in Table 2.

In our survey, we found 22 main objections from the general population to the nationwide introduction of the QR code-based system. They are summarized in Table 2. Figure 2 shows their frequency of occurrence.

From Figure 2 and Table 2, one can see that the social resistance to QR codes as tokens of digital COVID-19 vaccination certificates in Russia is huge and diverse. A lot of social fears and mistrustful behavioral patterns are fused in this resistance. That makes the Russian situation almost unprecedented. This unparalleled antipathy to QR codes in Russian society may be partly explained by the fact that a significant part of the Russian population regards the QR code-based system as a mere aimless technocratic innovation of the government and does not consider it as an electronic vehicle for transmitting vaccination-related information [81,82,83,84]. Besides, many Russians regard QR codes as a possible segregation system [85,86,87].

### 3.4. Limitations of the Study

1. It should be emphasized that the response rate was very low. The number of responses in the final sample set was equal to merely 6.8% of the number of invitations sent. It may be partly accounted for by (1) geographical differences of sentiments about vaccination across different Russian regions, (2) urban/rural differences, (3) capital (Moscow and St Petersburg)/provincial differences, (4) emotional and social fatigue caused by the pandemic, and (5) unwillingness to explain their viewpoint (low social activity, which may also be explained by significant COVID-19-related social burden). Therefore, our survey may not necessarily reflect the sentiments of the whole Russian population regarding vaccination in an exact manner.
2. The social network VKontakte places definite boundaries on age, occupation, mode of lifestyle, level of income, and social preferences (as any social network basically does). Therefore, the audience covered by VKontakte will be younger than that in another Russian social network, Odnoklassniki (“School Mates”). However, the technical capabilities of Odnoklassniki do not allow performing such research as ours.
3. An opinion poll carried on in a social network is a priori less representative than a poll performed “in the field,” (i.e., in public places). However, the pandemic situation dictates the demands of physical social distancing.
4. Persons with antivaccination sentiments are more active than supporters of vaccination, including activity in social networks [88,89,90]. Therefore, the percentage of vaccination antagonists in our research is likely to be higher than in the entire Russian society.

## 4. Conclusions

The current Russian vaccination campaign against SARS-CoV-2 is seriously endangered by several social factors, the main ones of which are:

- Concerns about excessive digitalization in the education, social welfare, and healthcare sectors instigated by the pandemic;
- Vexation over the lack of desire of officials to receive feedback from the general population regarding vaccination;
- Wide support for conspiracy beliefs; and
- Controversy over the nationwide QR code-based digital system of vaccination certification/verification.

We advance the following recommendations to raise the appeal of anti-SARS-CoV-2 vaccination to the Russian general population and trust in the authorities:

1. Authorities should make a shift from unrelenting pressure on society and threats of punishment for those who oppose vaccination to social encouragement for those who back it. They should also aim at raising the sentiments of moral responsibility among people to constitute a healthy society and finally stop the pandemic.
2. The vaccination campaign should be made more transparent, and the intentions of the government more straightforward. Official informational resources may provide much more detailed statistical information about the progression of vaccination and its success and drawbacks. Healthy self-criticism would increase the level of trust in federal and local authorities.
3. There should not be a technocratic approach to vaccination, which would see the success of the vaccination campaign in the methods and techniques applied, not in the final goal (e.g., QR codes only and nothing else). A flexible attitude may be more convenient and helpful. For those who deem QR codes and other forms of digital certification acceptable, they may be used. For those who oppose them on different grounds, a “classic” vaccination certificate may be issued by medical institutions with necessary data and authorization on a paper blank. Both should be accepted as valid forms of vaccination proof. Neither antagonism with society nor populism is productive in the COVID-19 containment program.
4. Inclusion of feedback from society and public discussions about vaccination should be performed. Collaboration with religious organizations, NGOs, NPOs, and prominent public figures may be useful here. Popular healthcare figures (e.g., reputed doctors and scientists) may be invited to official media for public discussions about the vaccination campaign. These figures can be determined in surveys and public opinion polls.
5. There should not be excessive digitalization connected to the vaccination campaign. Keeping hard (paper) copies of digital information about vaccination is important, as a serious power outage or Internet connection disruption may threaten the national database of vaccination.
6. A public disclosure of vaccine composition should be carried out in major media by inviting independent laboratories and experts. It does not mean going into medical and pharmacological particulars or disclosing commercial secrets. It means a measure aimed at eliminating fears of a considerable part of the population about being vaccinated with an “obscure liquid.” That measure may greatly reduce the number of supporters of anti-SARS-CoV-2 vaccination conspiracy beliefs in Russia.

Multimodality and flexibility are the main recipes for ensuring the success of anti-SARS-CoV-2 vaccination in Russia and, hopefully, a sooner end of the COVID-19 pandemic.

## Figures and Tables

**Table 1:** Main demographics and social preferences of the respondents in regard to anti-SARS-CoV-2 vaccination.

Student Distribution of the Sample Set Was Assumed, Confidence Interval (CI) = 95%, *p* = 0.05.	
Age	Range: 16–51 years old (y.o.) *; Mean: 29.1 ± 10.6 y.o.
Gender	2575 females (44.23% **)
At least cautious attitude towards vaccination	2454 (42.15%)
Active antivaccination attitude in regard to SARS-CoV-2, readiness to go to street protests	419 (7.20%)
Mean age of those with antivaccination attitude regarding SARS-CoV-2	21.4 ± 3.1 y.o
Confirmed antivaccination attitude in regard to any vaccines for any infectious diseases	43 (0.74%)
Concerns about COVID-19-related radical initiatives in education, social welfare, and healthcare public policy (excessive digitalization)	1972 (33.87%)
Annoyance because of the lack of administrative wish to notice feedback from the general population regarding the vaccination campaign (“they treat us as simpletons”). Annoyance because people who ask questions and wish to be heard are frequently marked by officials as “anti-vaxxers” who should be subdued or punished only	644 (11.06%)
Concern about pharmacological quality and safety of the Russian vaccines	928 (15.94%)
Preference of foreign vaccines to the Russian ones (readiness to get vaccinated by a foreign vaccine if it was available in Russia)	252 (4.33%)
Concern about equality in vaccine allocation	318 (5.46%)
Concerned about forthcoming introduction of QR code-based system of vaccination verification in Russia: - Those who may reconsider their wish to be vaccinated if the nationwide QR code-based system has been introduced - Those who regard the vaccination verification system of QR codes a part of a conspiracy	5064 (86.98%) 1548 (26.59%) 849 (14.58%)
Support for at least one conspiracist theory	1323 (22.72%)
Support for at least two conspiracist theories	1102 (18.93%)
Support for at least three conspiracist theories	1027 (17.64%)
Mean age of conspiracists	26.2 ± 6.6 y.o
Vaccinated with at least one dose or ready to be vaccinated	1006 (17.28%)
Those who think that vaccination must be voluntary	3625 (62.26%)
Those who think that vaccination must be compulsory	390 (6.70%)

* Responses outside the range are outliers. ** Hereinafter the percentage of the whole sample set suitable for work (i.e., the percentage of 5822 persons).

**Table 2:** The legend for Figure 2.

Designation	Objection
A	Concern about potential segregation of the Russian population (those with QR codes will enjoy full rights, and those without them will be castaways in society)
B	Complicated access to shops and markets
C	Concern about total control of personal relocation and whereabouts
D	Prevention of everyday commuting to workplaces in public transport
E	Moral unacceptability (human dignity cannot be downgraded to technical detail-labelling codes; humans cannot become “stamped animals”)
F	Prevention of freedom of movements within Russia by train and plane
G	Fears about total digitalization and accumulation of vital information about individuals by several governmental databases
H	Concern that QR codes disguise compulsory vaccination
I	New uncertain financial losses of many industries, new bankruptcies of firms
J	Excessive financial pressure among individuals
K	Excessive burden to small and middle-size businesses
L	Fears about endangered data privacy and possible fraud related to QR codes
M	Concern about full substitution of paper vaccination certificates by digital QR codes
N	Complicated access to public events
O	Concern about lack of protection of children who were issued QR codes
P	Concern about lack of financial protection, as some Russian banks started to integrate QR vaccination certificates in their online banking systems for individuals
Q	Religious unacceptability (“the seal of anti-Christ” and similar apocalyptic sentiments)
R	Fear of social tensions and conflicts
S	Unwillingness to buy smartphones and/or install QR code detection applications
T	Absence of the possibility to obtain a QR code on the basis of antibody tests
U	Concern about artificial intelligence taking over the control of our lives and freedoms
V	Aesthetical unacceptability (QR codes are not appealing)

## Data Availability

Available from the authors upon reasonable request.

## Supplementary Materials

The following supporting information can be downloaded at: https://www.mdpi.com/article/10.3390/ijerph19063387/s1. Questionnaire (translation from Russian into English).
