# Supplementary material for: Public Policy Measures to Increase Anti-SARS-CoV-2 Vaccination Rate in Russia
Source: Int J Environ Res Public Health. 2022 Mar 13;19(6):3387. doi: 10.3390/ijerph19063387 (PMC8955973; doi:10.3390/ijerph19063387)
Supplement: Supplementary file 1 [file ijerph-19-03387-s001.zip › ijerph-1594730-supplementary.pdf]

Questionnaire
(translated from Russian into English)

1. What are your attitudes regarding vaccination against COVID-19?
[response as a text]

2. Do you regard yourself a person with confirmed anti-vaccination attitude regarding different diseases?
[YES/NO]

3. If you are cautious about vaccination against COVID-19, please list your reasons (any number of reasons):
[response as a text]

4. If any foreign vaccines against COVID-19 were available in Russia, would you prefer them?
[YES/NO/DIFFICULT TO SAY]

5. Are you aware about the forthcoming introduction of nation-wide system of vaccination certification / verification based on QR codes?
[YES/NO]

6. If YES (Query 5), do you support this initiative?
[YES/NO/DIFFICULT TO SAY]

7. If YES (Query 5), please list your reasons (any number of reasons):
[response as a text]

8. Are you already vaccinated against COVID-19?
[YES/NO]

9. Do you think anti-COVID vaccination should be… (please continue)
[VOLUNTARY/COMPULSORY/DIFFICULT TO SAY]
